# Comparative Efficacy of Novel Versus Traditional Antiemetic Agents in Preventing Chemotherapy-Induced Nausea and Vomiting With Moderate or Highly Emetogenic Chemotherapy: A Systematic Review

**DOI:** 10.7759/cureus.72774

**Published:** 2024-10-31

**Authors:** Sally M Nashed, Rami Kamal A Morcos, Muhammad Atif, Abdullah Shehryar, Abdur Rehman, Reema Kumari, Safiyyah M Khan, Waleed Hassan, Muhammad Roshan Zeb, Talha A Zia, Syed Jameel

**Affiliations:** 1 Department of Forensic Medicine and Toxicology, Faculty of Medicine, Ain Shams University, Cairo, EGY; 2 Department of General Surgery, Ain Shams University Specialized Hospital, Cairo, EGY; 3 Department of General Surgery, Ministry of Health Holdings, Riyadh, SAU; 4 Department of General Medicine, Dow International Medical College, Karachi, PAK; 5 Department of Internal Medicine, Allama Iqbal Medical College, Lahore, PAK; 6 Department of Surgery, Mayo Hospital, Lahore, PAK; 7 Department of Medicine, Jinnah Postgraduate Medical Centre, Karachi, PAK; 8 Department of Research Methodology, California Institute of Behavioral Neurosciences and Psychology, Fairfield, USA; 9 Department of Medicine, Shaikh Zayed Hospital, Lahore, PAK; 10 Department of Pharmacy, Abbottabad University of Science and Technology, Abbottabad, PAK; 11 Department of Pharmacy, Shifa International Hospitals, Islamabad, PAK; 12 Department of Internal Medicine, King Edward Medical University, Lahore, PAK

**Keywords:** antiemetic agents, chemotherapy-induced nausea and vomiting (cinv), comparative efficacy, highly emetogenic chemotherapy (hec), moderate emetogenic chemotherapy (mec)

## Abstract

This systematic review critically evaluates the comparative efficacy of novel and traditional antiemetic agents in preventing chemotherapy-induced nausea and vomiting (CINV) in patients receiving moderate or highly emetogenic chemotherapy (MEC/HEC). The findings suggest that novel agents, such as netupitant/palonosetron (NEPA), olanzapine, and transdermal granisetron (GTDS), offer comparable or superior efficacy to traditional antiemetic regimens, including standard options like aprepitant combined with 5-HT₃ receptor antagonists and corticosteroids. These novel agents demonstrate strong effectiveness in controlling both acute and delayed CINV and offer practical advantages, such as simplified dosing regimens and improved patient adherence, particularly in resource-limited settings. Additionally, traditional regimens incorporating aprepitant remain effective in preventing CINV. However, the review highlights the need for more direct comparisons between novel and traditional agents, as well as further studies evaluating novel therapies in real-world clinical settings. Future research should focus on larger, long-term trials to better establish the role of novel antiemetic agents and optimize CINV management strategies.

## Introduction and background

Chemotherapy-induced nausea and vomiting (CINV) is one of the most distressing and debilitating side effects of cancer treatment, profoundly impacting patient quality of life and adherence to therapy [1]. Left inadequately controlled, CINV can lead to dehydration, malnutrition, electrolyte imbalance, and treatment delays, all of which contribute to poorer clinical outcomes. CINV occurs in both the acute phase, which manifests within 24 hours post-chemotherapy, and the delayed phase, which can last up to five days [2]. As chemotherapy regimens have evolved in their complexity and potency, the need for effective antiemetic strategies has become increasingly critical, particularly for patients receiving moderately or highly emetogenic chemotherapy (MEC and HEC) [3].

Over the past few decades, antiemetic management has advanced significantly with the development of new pharmacological agents. The advent of 5-hydroxytryptamine-3 (5-HT₃) receptor antagonists and neurokinin-1 (NK₁) receptor antagonists, combined with corticosteroids, has become the backbone of modern antiemetic protocols [4]. These regimens have shown promising efficacy in preventing acute and delayed CINV. However, with newer drugs like netupitant/palonosetron (NEPA) (a fixed combination of netupitant and palonosetron), fosaprepitant, and olanzapine, there is growing interest in understanding how these novel agents compare to traditional antiemetic therapies [5]. The introduction of long-acting granisetron transdermal system (GTDS) has further expanded the therapeutic landscape by offering a convenient and sustained-release alternative to oral or intravenous formulations [6].

Despite the progress, there remains variability in the management of CINV, particularly when comparing the effectiveness of newer antiemetic agents against established treatments. Additionally, variations in patient populations, types of cancer, chemotherapy protocols, and combinations of antiemetic drugs create challenges in determining the most effective strategies. This has driven ongoing research and meta-analyses aimed at refining antiemetic guidelines and improving outcomes for cancer patients.

The primary objective of this systematic review is to critically evaluate the comparative efficacy of novel versus traditional antiemetic agents in preventing CINV in patients receiving moderate or highly emetogenic chemotherapy. By synthesizing data from randomized controlled trials, network meta-analyses, and systematic reviews, this review aims to provide evidence-based insights into the relative effectiveness of newer regimens, such as NEPA, olanzapine, and transdermal granisetron, compared to more established protocols. In doing so, the review seeks to inform clinical decision-making, optimize CINV management, and identify gaps in the current literature that warrant further research.

## Review

Materials and methods

Search Strategy

Our search strategy was developed in accordance with the Preferred Reporting Items for Systematic Reviews and Meta-Analyses (PRISMA) guidelines [7] to ensure a comprehensive review of studies comparing the efficacy of novel and traditional antiemetic agents in preventing CINV in patients receiving MEC and HEC. We conducted systematic searches across multiple electronic databases, including PubMed, Medline, Embase, and the Cochrane Library, covering the period from database inception through September 2024.

We employed a combination of relevant Medical Subject Headings (MeSH) terms and keywords such as "chemotherapy-induced nausea and vomiting," "CINV prevention," "antiemetics," "neurokinin-1 receptor antagonists," "5-HT₃ receptor antagonists," and "olanzapine." Boolean operators ('AND', 'OR') were utilized to enhance the search sensitivity, yielding search strings like: "chemotherapy-induced nausea AND neurokinin-1 receptor antagonist," "5-HT₃ antagonist AND moderately emetogenic chemotherapy," and "palonosetron OR NEPA AND chemotherapy-induced nausea and vomiting." Additional articles were identified by screening the references of included studies, and relevant conference abstracts were reviewed to capture ongoing or unpublished trials. We limited the search to English-language, peer-reviewed articles, focusing on RCTs, systematic reviews, and meta-analyses.

Eligibility Criteria

The eligibility criteria for this systematic review were carefully established to ensure that only high-quality, relevant studies were included. We focused on peer-reviewed research articles, including RCTs, network meta-analyses, and systematic reviews, that specifically investigate the comparative efficacy of novel and traditional antiemetic agents in preventing CINV in patients undergoing MEC/HEC. The primary population of interest includes adult cancer patients receiving these chemotherapy regimens, and interventions involving both novel agents such as NEPA and olanzapine, as well as traditional treatments like 5-HT₃ receptor antagonists and NK₁ receptor antagonists.

Inclusion criteria were defined to ensure the relevance of the selected studies. We included studies that assess CINV prevention regimens, with outcomes measured in terms of complete response, no emesis, and patient quality of life. Only studies published in English between the inception of each database and September 2024 were considered. Non-peer-reviewed articles, animal studies, case reports, conference abstracts, and unpublished data were excluded from this review. Additionally, research focusing solely on pediatric populations or other conditions unrelated to CINV was excluded to maintain the review's focus on MEC/HEC in adult cancer patients.

Data Extraction

Our data extraction process was rigorously designed to ensure the accuracy and completeness of the data included in this systematic review on the comparative efficacy of novel versus traditional antiemetic agents in preventing CINV. Initially, two independent reviewers screened studies based on titles and abstracts, categorizing them as "relevant," "not relevant," or "potentially relevant." Studies classified as "potentially relevant" were then subjected to a full-text review to assess their eligibility based on predefined inclusion and exclusion criteria.

To maintain consistency and reliability, data from the eligible studies were extracted using a standardized form in Microsoft Excel (Microsoft, Redmond, Washington). This form captured key information, including study characteristics such as author, publication year, study design, population size, interventions, primary outcomes, and limitations. Any discrepancies between reviewers were resolved through discussion, with a third reviewer intervening when necessary to ensure consensus. This structured approach to data extraction facilitated a thorough and accurate synthesis of findings across the studies.

Data Analysis and Synthesis

Given the variability in study designs, populations, and interventions, we opted for a qualitative synthesis rather than a meta-analysis. Our data analysis focused on integrating and comparing the findings from each study to assess the relative efficacy of novel versus traditional antiemetic agents in preventing CINV across MEC/HEC. Key outcomes such as complete response rates, incidence of emesis, and patient-reported quality of life were extracted, and common patterns or discrepancies were identified across the studies.

This narrative approach enabled us to explore the nuanced effectiveness of different antiemetic regimens within varied clinical contexts, allowing for a detailed evaluation of the strengths and limitations of novel agents like NEPA, fosaprepitant, and GTDS. By synthesizing the findings, we provided a comprehensive overview of current evidence, discussed its implications for clinical practice, and highlighted areas where future research is necessary to optimize CINV management.

Results

Study Selection Process

**Figure 1:**
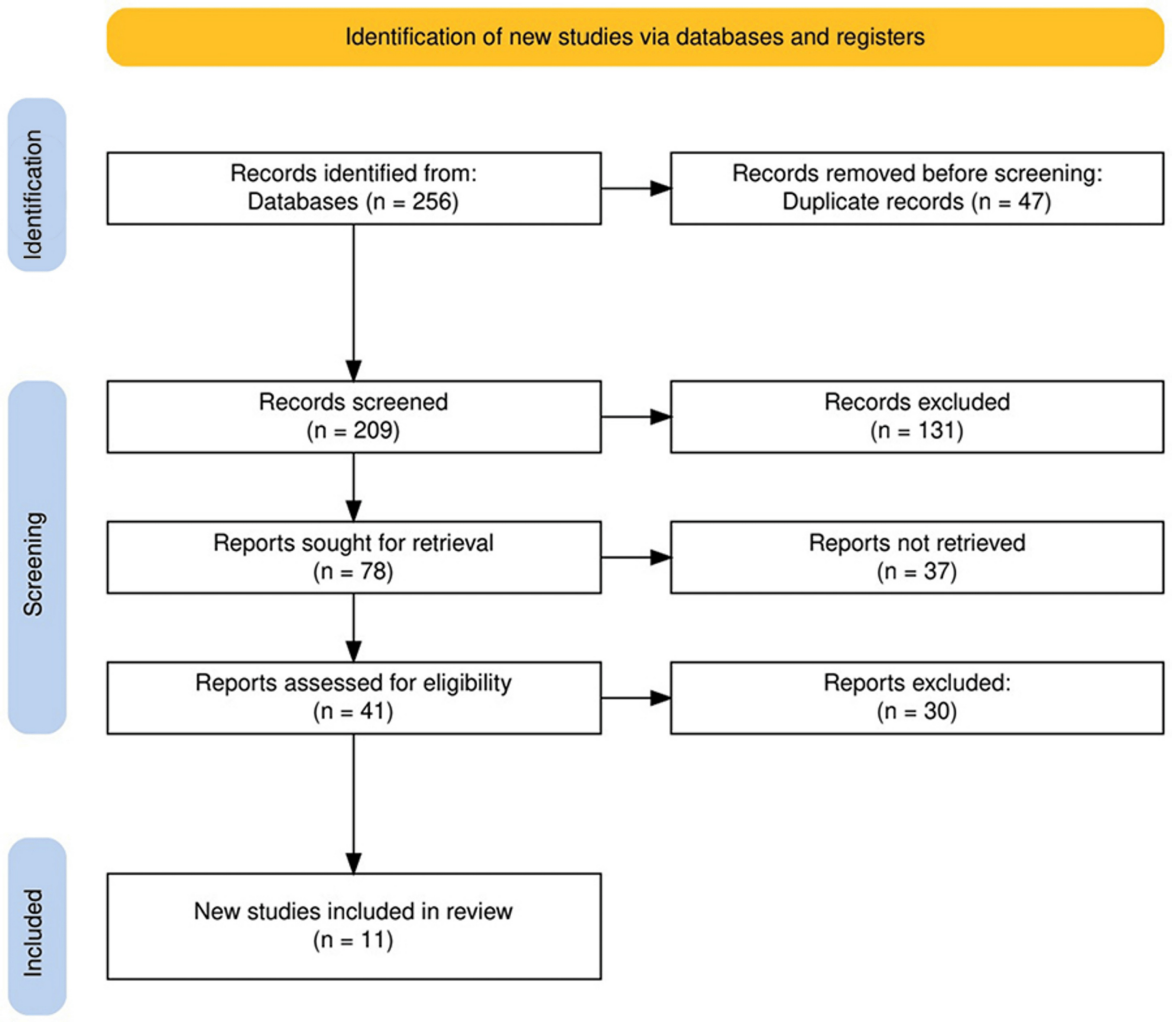
The PRISMA flow diagram of study selection process. PRISMA: Preferred Reporting Items for Systematic Reviews and Meta-Analyses.

The study selection process involved rigorous identification and screening of records from databases. Initially, 256 records were identified, but 47 duplicates were removed before screening. Out of the remaining 209 records, 131 were excluded based on relevance or eligibility criteria during the screening phase. The next step involved retrieving 78 reports, but 37 of these could not be obtained for assessment. Subsequently, 41 reports were evaluated for eligibility, and 30 were excluded for not meeting the necessary inclusion criteria. Ultimately, 11 new studies were included in the final review. This systematic approach ensured that only the most relevant and high-quality studies were incorporated into the analysis (Figure 1).

Characteristics of the Selected Studies

The selected studies, as illustrated in Table 1, encompass a range of designs, including RCTs, systematic reviews, and network meta-analyses, focusing on patients undergoing chemotherapy or suffering from cancer-related conditions. The population sizes vary across studies, with some involving as few as 20 participants, while others included over 22,000 patients. These studies examine the efficacy of various antiemetic regimens, such as NK₁ and 5-HT₃ antagonists, corticosteroids, and olanzapine, in preventing CINV. Key findings highlight the effectiveness of both traditional and newer combinations of these agents, with several studies identifying the most efficacious treatments in specific contexts (e.g., HEC or limited-resource settings). However, many studies also point to limitations, such as the need for larger trials, direct comparative studies, and further long-term evaluations to confirm the benefits observed in smaller or more controlled environments.

**Table 1:** Summary of key findings from selected studies on the efficacy of novel vs. traditional antiemetic agents in preventing chemotherapy-induced nausea and vomiting. NK₁: Neurokinin-1 receptor antagonist; 5-HT₃: 5-hydroxytryptamine-3 receptor antagonist; MEC: Moderately emetogenic chemotherapy; HEC: Highly emetogenic chemotherapy; NEPA: Fixed combination of netupitant and palonosetron; GTDS: Granisetron transdermal system; DEX: Dexamethasone; RCT: Randomized controlled trial; QoL: Quality of life.

Author	Year	Study Design	Population Size	Key Findings	Limitations
Piechotta V et al. [8]	2021	Network Meta-Analysis	Adults with solid or haematological cancers	NK₁, 5-HT₃ antagonists, and corticosteroids effective; fosnetupitant + palonosetron most efficacious in HEC.	Imprecision and inconsistency in network meta-analysis results.
Filetti M et al. [9]	2023	Network Meta-Analysis	22,228 patients	4-drug regimens with olanzapine most effective; 3-drug in limited resources.	Need for direct comparative studies for new regimens.
Zelek L et al. [10]	2021	Randomized Controlled Trial	202 patients	NEPA noninferior to aprepitant with potential benefits in MEC.	Further comparative studies needed.
Minatogawa H et al. [11]	2020	Phase III Randomized Controlled Trial	262 patients	DEX sparing non-inferior to traditional; ongoing study.	Further evidence needed for DEX sparing viability.
Chow R et al. [12]	2018	Systematic Review and Meta-Analysis	Patients undergoing chemotherapy	Palonosetron superior to other 5-HT3RAs for specific endpoints.	Further studies needed for broader clinical significance.
Micha JP et al. [13]	2016	Randomized Controlled Trial	20 patients	Comparable efficacy of fosaprepitant and aprepitant in gynecologic cancer CINV.	Larger studies needed for more robust data.
Seol YM et al. [14]	2016	Randomized Controlled Trial	196 patients	GTDS comparable to palonosetron with high patient satisfaction.	Larger population studies needed for long-term outcomes.
Rapoport BL et al. [15]	2010	Randomized Controlled Trial	848 patients	Aprepitant more effective than control across MEC regimens.	Continued evaluation needed in other patient populations.
Yeo W et al. [16]	2009	Randomized Controlled Trial	124 assessable patients	Aprepitant reduces rescue medication use and improves QoL compared to standard regimen.	Larger studies needed to confirm findings in other populations.
Boccia RV et al. [17]	2010	Randomized Controlled Trial	582 patients	GTDS non-inferior to oral granisetron for multi-day chemotherapy.	Further studies on long-term tolerability and adherence needed.
Schmoll HJ et al. [18]	2006	Randomized Controlled Trial	Significant RCT results	Aprepitant superior in all phases compared to ondansetron + dexamethasone.	More studies on long-term outcomes and different populations needed.

Discussion

This systematic review highlights the comparative efficacy and safety of novel versus traditional antiemetic agents in preventing CINV in patients undergoing MEC/HEC. Across the studies, novel agents such as NEPA (a fixed combination of netupitant and palonosetron), olanzapine, and GTDS) were found to be effective alternatives to traditional regimens that primarily involved NK₁ and 5-HT₃ receptor antagonists. Notably, Filetti M et al. [9] demonstrated that a 4-drug regimen with olanzapine provided the highest probability of complete response in preventing CINV, while 3-drug regimens with olanzapine were also beneficial in resource-limited settings. Piechotta V et al. [8] further supported the efficacy of NK₁ receptor antagonists combined with 5-HT₃ antagonists and corticosteroids, identifying fosnetupitant plus palonosetron as the most efficacious regimen in HEC. Other studies, such as those by Zelek L et al. [10] and Micha JP et al. [13], confirmed the non-inferiority of NEPA and fosaprepitant, respectively, compared to traditional aprepitant regimens in MEC.

While many novel regimens demonstrated superiority or non-inferiority, some traditional agents, such as aprepitant, remain highly effective. For example, Schmoll HJ et al. [18] reported that an aprepitant regimen was superior to ondansetron plus dexamethasone in controlling CINV across all phases of high-dose cisplatin therapy. Similarly, Rapoport BL et al. [15] highlighted the consistent efficacy of aprepitant across various MEC regimens. However, studies like those by Chow R et al. [12] showed that while palonosetron was statistically superior to other 5-HT₃ antagonists, its clinical significance was limited to specific endpoints. Overall, the review indicates that novel agents like NEPA, olanzapine, and GTDS are not only effective but may also offer improved convenience and patient adherence compared to traditional regimens [19], though continued research is needed to solidify their roles in standard antiemetic protocols.

The findings of this systematic review largely align with existing clinical guidelines from major oncology bodies such as the Multinational Association of Supportive Care in Cancer/European Society for Medical Oncology (MASCC/ESMO), the American Society of Clinical Oncology (ASCO), and the National Comprehensive Cancer Network (NCCN), which recommend the use of neurokinin-1 (NK₁) receptor antagonists, 5-hydroxytryptamine-3 (5-HT₃) receptor antagonists, and corticosteroids for the prevention of CINV in patients receiving MEC and HEC. Studies included in this review, such as those by Piechotta V et al. [8] and Filetti M et al. [9], support these guidelines by demonstrating the efficacy of combination regimens involving NK₁ and 5-HT₃ antagonists in preventing both acute and delayed CINV. Furthermore, the review highlights that novel agents like NEPA and olanzapine offer effective alternatives, consistent with recent updates in the MASCC/ESMO guidelines, which acknowledge these newer agents as valid options. The non-inferiority of NEPA, as reported by Zelek L et al. [10], is particularly relevant given its simplified dosing, which aligns with current efforts to enhance patient adherence through easier administration protocols.

However, while many of the findings support current recommendations, certain gaps in the literature remain. For example, although Chow R et al. [12] demonstrated the statistical superiority of palonosetron over other 5-HT₃ antagonists, its limited clinical significance across broad endpoints suggests that current guidelines may need to refine their recommendations for specific clinical scenarios. Similarly, the high efficacy of olanzapine in the studies by Filetti M et al. [9] and Minatogawa H et al. [11] suggests that guidelines might increasingly prioritize this agent in future recommendations, particularly in resource-limited settings where cost-effectiveness is crucial [20].

The findings from this review have significant practical implications for the management of CINV in clinical practice. Novel antiemetic agents such as NEPA, olanzapine, and GTDS offer promising alternatives to traditional regimens, particularly for patients undergoing MEC/HEC [21]. NEPA, with its single-dose administration, simplifies dosing regimens, potentially enhancing patient adherence and reducing the complexity of antiemetic management [22]. This is particularly beneficial in resource-limited settings, where access to healthcare and frequent administration of medications may pose challenges. GTDS, which provides sustained release over seven days, also offers a convenient option for patients, reducing the need for daily dosing and minimizing disruptions to treatment schedules [23]. These novel agents, with their favorable side effect profiles and ease of use, could significantly improve the quality of life for cancer patients, streamline clinical workflows, and support better adherence to chemotherapy regimens, ultimately contributing to improved clinical outcomes.

This systematic review boasts several strengths, including a comprehensive search strategy across multiple databases and adherence to PRISMA guidelines, ensuring a thorough and transparent approach to study selection and data extraction. The inclusion of diverse study designs, such as RCTs, network meta-analyses, and systematic reviews, enhances the robustness of the findings and provides a well-rounded comparison of both novel and traditional antiemetic agents in preventing CINV. The rigorous inclusion criteria ensured that only high-quality, peer-reviewed studies were considered, adding to the credibility of the conclusions drawn.

However, there are some limitations that must be acknowledged. The heterogeneity of the included studies, particularly regarding variations in chemotherapy regimens, patient populations, and outcome measurements, may introduce challenges in comparing results across studies. Additionally, the reliance on published literature excludes potentially valuable insights from unpublished trials or grey literature. Certain studies also had small sample sizes or lacked blinding, which could introduce bias and limit the generalizability of the findings.

Despite the wealth of data reviewed, several gaps in the literature remain. A key unresolved issue is the lack of head-to-head comparisons between novel and traditional antiemetic agents, particularly in real-world clinical settings. While some novel agents like NEPA and GTDS have shown promise, there is limited evidence directly comparing their efficacy with established agents such as aprepitant and ondansetron across diverse cancer populations and chemotherapy regimens [24]. Additionally, certain populations, such as those undergoing high-dose chemotherapy or those in resource-limited settings, are underrepresented in the current research. These gaps highlight the need for further investigation to optimize antiemetic strategies tailored to specific patient groups and treatment protocols [25].

Future research should focus on conducting larger RCTs to provide robust evidence on the long-term efficacy and safety of novel antiemetic agents like olanzapine and NEPA. Comparative studies between novel and traditional regimens are also essential, particularly in real-world clinical practice, where patient adherence and treatment tolerability are key considerations. Additionally, further exploration of the role of olanzapine in resource-limited settings could yield valuable insights, as could research on the long-term tolerability of transdermal formulations like GTDS, which offer more convenient dosing options. By addressing these areas, future studies can help refine CINV prevention guidelines and improve patient outcomes.

## Conclusions

This systematic review highlights the significant potential of novel antiemetic agents, such as NEPA, olanzapine, and GTDS, in improving the management of chemotherapy-induced nausea and vomiting (CINV), particularly in patients undergoing MEC/HEC. These agents have demonstrated comparable or superior efficacy to traditional regimens while offering benefits like simplified dosing and enhanced patient adherence. The findings suggest that incorporating these novel agents into clinical practice could optimize CINV prevention and improve patient outcomes, especially in diverse settings. However, further research is needed to confirm their long-term efficacy and safety across broader patient populations and chemotherapy regimens. Continued exploration of novel agents will be essential in shaping future guidelines and strategies for the effective prevention of CINV.
